# Clinical utility of FAI and SHBG in differentiating PCOS from anovulatory cycles in adolescent girls

**DOI:** 10.3389/fped.2025.1581060

**Published:** 2025-08-08

**Authors:** Emre Özer, Demet Taş, Seçil Çakır Gündoğan, Mehmet Boyraz, Fatih Gürbüz

**Affiliations:** ^1^ Department of Pediatric Endocrinology, Ankara Bilkent City Hospital, Ankara, Türkiye; ^2^ Department of Pediatrics, Division of Adolescent Health, Faculty of Medicine, Ankara Yıldırım Beyazıt University, Ankara, Türkiye; ^3^ Division of Adolescent Health, Department of Pediatrics, Ankara Bilkent City Hospital, Ankara, Türkiye; ^4^ Department of Pediatric Endocrinology, Faculty of Medicine, Ankara Yıldırım Beyazıt University, Ankara, Türkiye

**Keywords:** free androgen index, menstrual irregularity, LH-FSH, oligomenorrhea, polycystic ovary syndrome, sex hormone binding globuline

## Abstract

**Introduction:**

Menstrual irregularities are common in adolescents, often linked to anovulatory cycles. This study aims to establish diagnostic cut-off values for Polycystic Ovary Syndrome (PCOS) and differentiate it from anovulatory dysfunction in adolescents, while evaluating the diagnostic sensitivity of the Free Androgen Index (FAI) and Sex Hormone Binding Globulin (SHBG).

**Methods:**

The study included 305 adolescents with oligomenorrhea at a tertiary center. Statistical analyses were performed, and Receiver Operating Characteristic (ROC) curves were used to assess diagnostic performance.

**Results:**

Of the 305 patients, 229 (75%) had anovulatory cycles, and 36 (11.8%) were diagnosed with PCOS. Mean FAI values were 3.5 ± 2 in anovulatory cycles, 8.0 ± 5 in PCOS, and 8.3 ± 4 in hyperinsulinism (*p* < 0.001). FAI showed significant positive correlations with HOMA-IR (*r* = 0.389, *p* < 0.001) and BMI *z*-score (*r* = 0.499, *p* < 0.001). ROC analysis identified an LH threshold of 9.7 U/L and an LH/FSH ratio of 2.62 as predictive markers for PCOS.

**Discussion:**

Anovulatory cycles are the leading cause of menstrual irregularities in adolescents. While hyperandrogenism is crucial for PCOS diagnosis, elevated FAI levels in PCOS are also observed in hyperinsulinemia and obesity. PCOS is more prevalent in obese adolescents, which limits the diagnostic reliability of FAI. Lower SHBG levels in hyperinsulinemic obese adolescents further complicate FAI interpretation, underscoring the significant impact of glucose and insulin metabolism on these markers. Therefore, a comprehensive diagnostic approach, including androgen levels, LH/FSH ratio, SHBG, FAI, and ovarian ultrasound, is essential for accurate PCOS diagnosis in adolescent girls.

## Introduction

Abnormal uterine bleeding (AUB) is a common problem among adolescents, with oligomenorrhea often resulting from anovulatory cycles caused by the immaturity of the hypothalamic-pituitary-ovarian (HPO) axis (1–4). Polycystic Ovary Syndrome (PCOS), a prevalent endocrine disorder, affects 3.4%–19.6% of adolescents and is characterized by hyperandrogenism and oligomenorrhea. The pathogenesis of PCOS is multifactorial and not fully elucidated, characterized by excess androgen production involving dysregulation of ovarian steroidogenesis, and significant alterations in insulin metabolism. However diagnosing PCOS in adolescents differs significantly from adults due to the physiological immaturity of the HPO axis. This immaturity can result in transient ovulatory dysfunction and mild hyperandrogenism that may mimic PCOS. Moreover, clinical features such as hirsutism and acne in adolescents may reflect normal pubertal development rather than true pathological androgen excess, complicating the diagnostic process. Ultrasound assessment based on the Rotterdam criteria defined by the presence of ≥12 follicles measuring 2–9 mm in diameter or an ovarian volume >10 ml in at least one ovary is not recommended for diagnosing PCOS in adolescents. This is due to the fact that such findings may reflect normal physiological changes related to the immaturity of the axis, rather than pathologic conditions. Consequently, USG findings in this age group must be interpreted with caution and are not considered definitive for diagnosis (5–10). Genome-Wide Association Studies (GWAS), have identified several candidate genes associated with PCOS susceptibility, including *DENND1A* (DENN Domain-Containing Protein 1A), *LHCGR* (Luteinizing Hormone/Chorionic Gonadotropin Receptor), and *INSR* (Insulin Receptor) (11, 12). These genetic insights highlight the complex interplay of hormonal, metabolic, and genetic factors in PCOS development. Adolescents with PCOS are at an increased risk of developing long-term health complications, such as metabolic syndrome, infertility, endometrial cancer, and psychological disorders, including anxiety and depression. Given these risks, establishing accurate diagnostic criteria for PCOS during adolescence is critical to enable early intervention and prevent adverse outcomes. The Free Androgen Index (FAI), calculated as [total testosterone (nmol/L)/Sex Hormone Binding Globulin (SHBG) (nmol/L)] × 100, is a composite index that provides a more comprehensive assessment of bioavailable androgens compared to individual laboratory parameters such as total testosterone or SHBG alone. Unlike total testosterone, which measures both bound and unbound fractions, FAI accounts for the proportion of testosterone that is biologically active by incorporating SHBG, a key regulator of androgen bioavailability. However, obesity and hyperinsulinemia, which are frequently observed in adolescents with PCOS, exacerbate diagnostic challenges by altering key biomarkers such as the FAI and SHBG, making it difficult to distinguish PCOS from other anovulatory conditions.

In this study, we aimed to determine diagnostic cut-off values that can effectively differentiate PCOS from anovulatory dysfunction in adolescents. Additionally, we sought to evaluate the sensitivity of the FAI and SHBG in diagnosing PCOS, particularly in adolescents with complicating factors such as obesity and hyperinsulinemia.

## Materials and methods

### Study design and patient selection

This retrospective study was conducted at “Ankara Bilkent City” Hospital, a tertiary care center in Ankara, Türkiye, between August 2019 and March 2023. It included 305 adolescent girls who presented with oligomenorrhea and were at least 2 years post-menarche. Oligomenorrhea was defined as irregular menstrual cycles, with diagnostic criteria varying by postmenarcheal year: >90 days in the first year, >60 days in the second year, >45 days in the third year, and >38 days in the fourth year and beyond (13).

### Classification and exclusion of other etiologies

All patients were evaluated in accordance with the FIGO PALM-COEIN classification system for AUB, which includes structural and non-structural causes (polyp, adenomyosis, leiomyoma, malignancy/hyperplasia, coagulopathy, ovulatory dysfunction, endometrial disorders, iatrogenic, and not otherwise classified) (7).

• Patients diagnosed with PCOS or hyperinsulinemia were considered part of the ovulatory dysfunction (O)group, in line with the PALM-COEIN framework.
• Patients without any identifiable PALM or COEIN etiology, but who met the definition of oligomenorrhea and did not meet diagnostic criteria for PCOS or hyperinsulinemia, were categorized as having anovulatory cycles (14).
• To improve diagnostic accuracy and exclude confounding conditions, patients with chronic illnesses (*n* = 5), hypogonadism (*n* = 4), non-classic congenital adrenal hyperplasia (*n* = 2), prolactinoma (*n* = 2), and one patient with an estradiol-secreting ovarian cyst were excluded from the statistical analysis.

**Figure 1:**
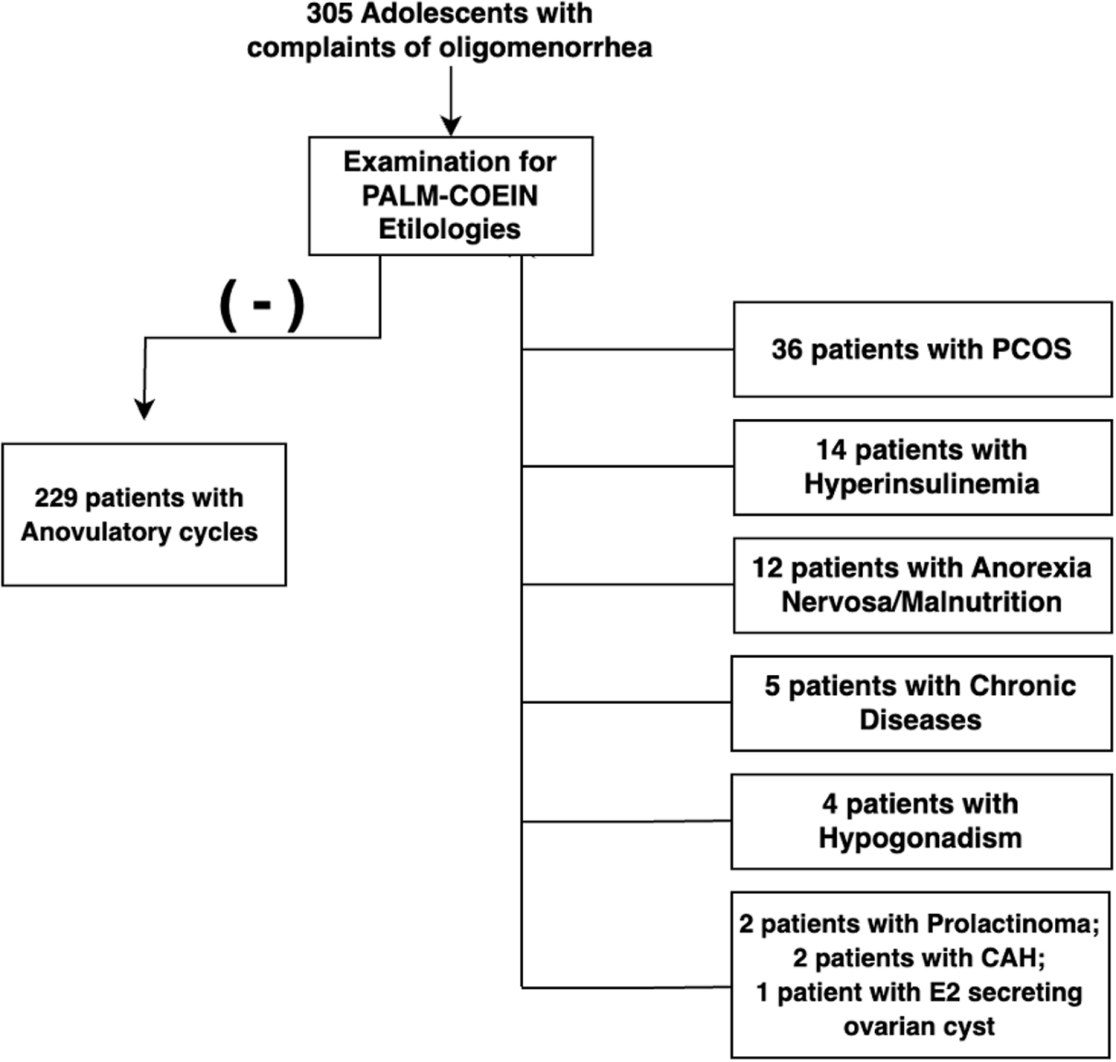
Flowchart of the patient selection.

All evaluations were performed at the same institution to ensure consistency in diagnostic procedures and enhance dataset homogeneity. A team of four pediatric endocrinologists and one adolescent health specialist reviewed all data. A detailed flowchart illustrating the patient selection is presented in Figure 1.

### Diagnosis of PCOS

PCOS was diagnosed based on adolescent-specific criteria and required both:

1. Oligomenorrhea, and
2. Evidence of hyperandrogenism, either:
  (a) Biochemical: Total testosterone level ≥50 ng/dl, or
  (b) Clinical: Moderate-to-severe hirsutism (modified Ferriman-Gallwey score ≥16) (13, 15).

### Hyperinsulinemia and obesity

Obesity and insulin resistance are known contributors to ovulatory dysfunction (16, 17). In this study, participants with a HOMA-IR value greater than 4 and fasting insulin levels exceeding 25 mU/L were classified as hyperinsulinemic. Although these metabolic indicators are associated with an increased risk of PCOS, participants who did not meet the diagnostic criteria were not categorized as having the syndrome.

The **HOMA-IR** index was calculated using the formula: (FastingInsulin[mU/L]×FastingGlucose[mg/dl])/405 (18, 19).

**Free Androgen Index:** FAI was calculated as: (TotalTestosterone(nmol/L)/SHBG(nmol/L))×100 (20).

### Sample collection and analysis

Blood samples were collected during the follicular phase of the menstrual cycle, when possible. For participants using oral contraceptive pills, samples were obtained either prior to initiating the medication or during the pill-free interval to minimize hormonal interference. LH, FSH, total testosterone, estradiol (E2), and insulin were analyzed by chemiluminescence immunoassay (Siemens Healthineers, Erlangen, Germany). SHBG was measured using the IMMULITE 2000 XPi system (Siemens, Cary, NC, USA). AMH was analyzed by ELISA (VIDAS AMH, bioMérieux, Marcy l'Étoile, France). Plasma glucose was determined by enzymatic colorimetric method. The levels of steroid hormones were measured using the Siemens Atellica Solution IM, an automated chemiluminescent immunoassay system platform. All samples were processed according to the manufacturer's protocols, with appropriate quality control measures implemented to validate assay performance.

### Statistical methods

The data were analyzed using SPSS 22 software (Statistical Package for Social Sciences; SPSS Inc., Chicago, IL). Descriptive statistics were used to report categorical data as “*n*”, and percentages, while continuous data were presented as means ± standard deviation (±SD). Pearson's chi-square test was used to compare categorical variables between groups. The normality of continuous variables was assessed using the Kolmogorov–Smirnov test. The Mann–Whitney *U*-test was used to compare two groups, and the Kruskal–Wallis test was used for comparisons between more than two groups. Spearman correlation test was used to examine the relationship between continuous variables. Receiver operating characteristic (ROC) curves were plotted to evaluate the diagnostic performance of various parameters. The level of statistical significance was set at *p* < 0.05 for all analyses.

## Results

### Patient characteristics

The mean age at diagnosis was 15.3 ± 1.5 years, with the onset of complaints occurring at 13.6 ± 1.6 years on average. The mean age of menarche was 12.5 ± 1.2 years, and patients reported the onset of complaints occurring approximately 1.0 ± 1.3 years after menarche. On average, 1.7 ± 1.5 years elapsed between the onset of complaints and presentation to the center.

Out of the 305 patients included in the study: 229 (75%) diagnosed with anovulatory cycles, 36 (11.8%) with PCOS, *n*: 14 (4.6%) with obesity and hyperinsulinism, *n*: 12 (3.9%) with severe malnutrition and others (5 patients had chronic diseases, 4 had hypogonadism, 2 had congenital adrenal hyperplasia, and 1 patient presented with an estradiol-secreting ovarian cyst. Additional demographic and clinical characteristics are summarized in Table 1.

**Table 1:** Characteristics of the patients.

Menstrual pattern	Oligomenorrhea	*n*; (%)
		264 (86.6%)
	Secondary amenorrhea (over 6 months)	41 (13.4%)
Excessive menstrual bleeding	Normal	238 (78%)
	Present	67 (22%)
BMI SDS, mean ± SS, (*n*)		0.6 ± 1.6 (277)
Hirsutism	Present	56 (18.4%)
	Normal	249 (81.6%)
Suprapubic ultrasonography	Performed	277 (90.8%)
	Not performed	28 (9.2%)
OCS treatment	Received	83 (27.2%)
	Not-received	222 (72.8%)

BMI, body mass index; m-FG, modified Feriman-Gallwey; OCS, oral contraceptive.

### Comparison of PCOS, anovulatory cycles, and hyperinsulinemia groups

Hirsutism showed a significant difference between the PCOS and anovulatory groups (*p* = 0.007). Significant differences were also observed in total testosterone and DHEAS levels between the PCOS group and the other groups (*p* < 0.001). Detailed analyses are presented in Table 2.

**Table 2:** Analysis of the patients with PCOS, anovulatuary cycles, and hyperinsulinism.

Variable		PCOS (*n* = 36)	Anovulatuary (*n* = 229)	Hyperinsulinism (*n* = 14)	*p* *
Age at the onset of complaints		13.7 ± 1.5 (35)	13.5 ± 1.6 (220)	13.1 ± 1.7 (12)	0.374
Age at menarche		12.5 ± 1.2 (35)	12.5 ± 1.2 (221)	12.2 ± 1.4 (13)	0.835
Time between the menarche and the onset of complaints (years)		1.3 ± 1.4 (35)	0.9 ± 1.2 (214)	0.9 ± 1.0 (12)	0.645
The time between the onset of complaints and the patient's application, (years)		2.1 ± 1.6 (35)	1.7 ± 1.5 (220)	1.7 ± 1.3 (12)	0.352
Menstrual pattern	Oligomenorrhea	31 (86.1%)	204 (89.1%)	13 (92.9%)	0.793**
	Secondary amenorrhea (over 6 months)	5 (13.9%)	25 (10.9%)	1 (7.1%)	
BMI, SDS		1.2 ± 1.5^b^ (36)	0.6 ± 1.3^c^ (203)	2.9 ± 0.6^a^ (13)	**<0.001**
Hirsutism		14 (38.9%^b^)	38 (16.6%^a^)	3 (21.4%^a,b^)	**0.007** **
m-FG score		17.1 ± 4.5^a^ (14)	9.8 ± 1.6^b^ (37)	10.0 ± 1.0^a,b^ (3)	**<0.001**
Insulin, mU/L		18.6 ± 12.1^b^ (22)	14.3 ± 6.2^b^ (89)	47.5 ± 48.1^a^ (12)	**<0.001**
HOMA-IR		4.1 ± 2.7^b^ (21)	3.0 ± 1.4^b^ (89)	11.0 ± 12.4^a^ (12)	**<0.001**
FSH, U/L		6.4 ± 1.5 (36)	6.1 ± 2.3 (187)	6.8 ± 2.3 (13)	0.332
LH, U/L		13.5 ± 8.5 (36)	10.5 ± 8.2 (187)	9.8 ± 6.0 (13)	0.103
LH/FSH		2.1 ± 1.3 (36)	1.7 ± 1.1 (187)	1.3 ± 0.6 (13)	0.134
Total Testosteron, ug/L		54.8 ± 20.7^b^ (36)	30.0 ± 9.5^a^ (177)	29.3 ± 8.6^a^ (12)	**<0.001**
DHEAS, ug/dl		291.9 ± 118.2^b^ (34)	199.3 ± 95.6^a^ (179)	163.6 ± 63.7^a^ (12)	**<0.001**
SHBG, nmol/L		29.2 ± 14.5^b^ (28)	42.1 ± 24.3^c^ (147)	12.2 ± 4.4^a^ (13)	**<0.001**
FAI		8.0 ± 5.1^a^	3.5 ± 2.7^b^	8.3 ± 4.1^a^	<0.001
OCS treatment	Received	13 (36.1%)	61 (26.6%)	5 (35.7%)	0.412**
	Not-received	23 (63.9%)	168 (73.4%)	9 (64.3%)	

* Kruskal Wallis analysis.

** Chi-square analysis performed.

^a,b,c^ The group from which the difference originates. PCOS, polcyctic ovarian syndrome; BMI, body mass index; m-FG, modified Feriman-Gallwey; HOMA-IR, homeostasis model assessment—insulin resistance; FSH, follicular stimulating hormone; LH, luteinizing hormone; SHBG, sex hormone binding globulin; DHEA-S, dehydroepiandrosterone-sulfate; OCS, oral contraceptive.

Bold values indicate statistical significance at *p* < 0.05.

### Free androgen index analysis

FAI was evaluated as a diagnostic criterion by comparing data among patients with anovulatory cycles, PCOS, and hyperinsulinemia. The mean FAI values were 3.5 ± 2 in the anovulatory cycle group, 8.0 ± 5 in the PCOS group and 8.3 ± 4 in the hyperinsulinemia group. A significant difference was observed between the anovulatory group and the PCOS/hyperinsulinemia groups (*p* < 0.001). Additionally, FAI showed significant positive correlations with HOMA-IR (*r* = 0.389; *p* < 0.001) and BMI *z*-score v(*r* = 0.499; *p* < 0.001).

### Receiver operating characteristic analysis

The diagnostic performance of various parameters for predicting PCOS was assessed. An LH threshold of 9.7 U/L showed a sensitivity of 63.9%, specificity of 59.9%, and statistically significant predictive capability. An LH/FSH ratio threshold of 2.62 had a sensitivity of 38.9%, specificity of 86.8%, and was found to be a statistically good predictor.

**Figure 2:**
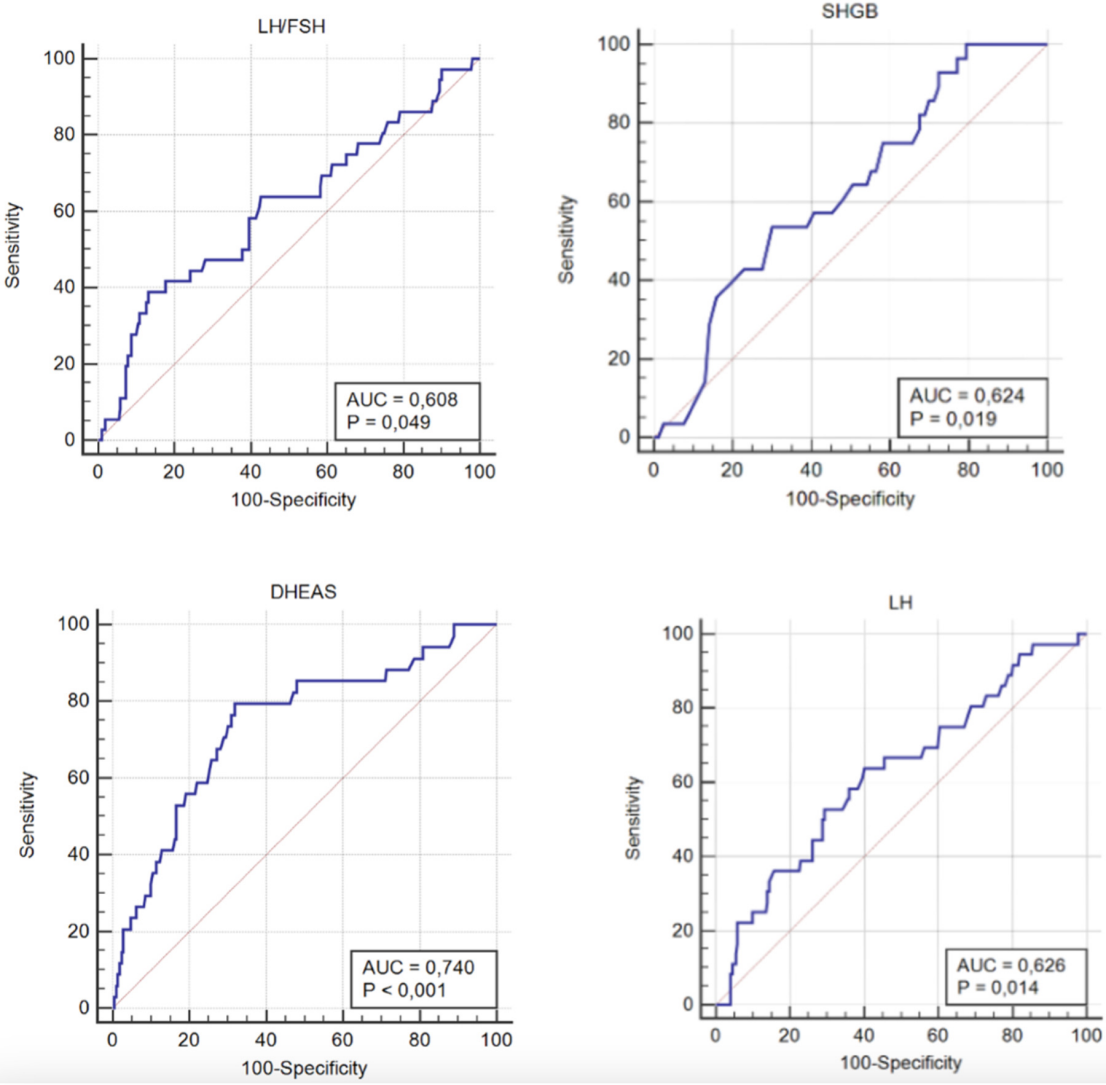
ROC curve analyses of the LH/FSH ratio, SHBG, DHEA-S, and LH levels for the diagnosis of PCOS.

The diagnostic performance of SHBG, LH, the LH/FSH ratio, and DHEA-S is illustrated by the ROC curves presented in Figure 2. Corresponding Youden indices are provided in Table 3.

**Table 3:** ROC analysis of the patient's data to predict PCOS.

Marker (cut-off)	Area	*p*	%95 safety zone		Sensitivity	Specificity	Youden index	PPV	NPD
			Lower limit	Upper limit					
SHGB ≤ 23 nmol/L	0.624	0.019	0.553	0.692	53.6	70.0	0.236	22.7	90.2
FSH > 5.3 U/L	0.561	0.164	0.498	0.623	83.3	36.2	0.195	17.5	93.0
LH > 9.7 U/L	0.626	0.014	0.563	0.685	63.9	59.9	0.238	20.5	91.1
LH/FSH > 2.62	0.608	0.049	0.545	0.668	38.9	86.8	0.257	32.6	89.7
E2 > 54 pg/ml	0.560	0.224	0.494	0.625	62.5	54.3	0.168	18.0	90.0
DHEA-S > 226 ug/dl	0.740	<0.001	0.680	0.794	79.4	68.1	0.475	28.7	95.3

SHBG, sex hormone binding globulin; FSH, follicular stimulating hormone; LH, luteinizing hormone; E2, estradiol; DHEA-S, dehydroepiandrosterone-sulfate; PPV, positive predictive value; NPV, negative predictive value.

## Discussion

The primary finding of our study is that FAI values were closely matched between adolescents with PCOS and those with hyperinsulinemia and obesity, raising concerns about the reliability of FAI as a diagnostic tool for PCOS. Additionally, SHBG levels were significantly lower in hyperinsulinemic/obese adolescents with oligomenorrhea compared to those with PCOS, suggesting that SHBG alone may not be sufficient to reliably distinguish PCOS, particularly in the presence of hyperinsulinemia.

In healthy adolescents, the average age of menarche typically ranges between 12 and 13 years. Our findings align with Zuchelo et al.'s meta-analysis, which reported that adolescents with PCOS have a similar age of menarche compared to non-PCOS adolescents (21). This suggests that oligomenorrhea is unrelated to the timing of menarche. However, adolescents' awareness of menstrual cycles and disorders can vary due to social and educational factors, often delaying their seeking of medical help (22). The average duration of symptoms in our cohort underscores the need for greater awareness and education regarding menstrual health. Menstrual irregularities, particularly menorrhagia, may serve as the first clinical sign of an underlying bleeding disorder. Given that coagulopathy has been reported in up to 20% of adolescents presenting with heavy menstrual bleeding, its exclusion remains essential during the diagnostic process (23).

The prevalence of PCOS among adolescents ranges from 4% to 19.6% (5, 7, 8). While our findings align with the reported range, it is important to note that our data are based on a hospital-based population presenting with oligomenorrhoea, which may not reflect the general community prevalence. The pathophysiology of PCOS involves functional ovarian hyperandrogenism (FOH), which increases GnRH pulse frequency and subsequently elevates LH levels. The LH/FSH ratio has been widely investigated as a diagnostic marker for PCOS, with studies reporting varying cut-off values. For instance; Le et al. reported a mean LH/FSH ratio of 2.08 in 441 PCOS patients, with a cut-off >1.33 showing 65.76% sensitivity and 95.24% specificity (24). A Chinese study involving 111 PCOS patients found a mean LH/FSH ratio of 1.87 ± 0.76 (25). Khashchenko et al. proposed an LH/FSH ratio cut-off >1.23, with sensitivity >75% and specificity >83% in 130 adolescents with PCOS (26). Unlike other studies, our cohort exclusively included adolescents presenting with oligomenorrhea, providing greater specificity for distinguishing PCOS from anovulatory dysfunction in this population. While the LH/FSH ratios in our PCOS patients were higher than those in previous studies, we observed no significant difference between the PCOS and anovulatory groups. Testosterone levels remain a controversial diagnostic criterion for PCOS, with varying cut-offs reported across studies. In a recent review/meta-analysis Zuchelo et al. reported patients that biochemical hyperandrogenism can be defined by serum testosterone levels over 48 ng/dl (21), while Le et al. reported an average testosterone level of 36.9 ± 25 ng/dl in PCOS patients (24). Khashchenko et al. found that up to one-third of PCOS patients had testosterone levels below 50 ng/dl which was the cut-off we used for biochemical hyperandrogenism (26). Hirsutism also varies among populations. For example, in Middle Eastern populations, a modified Ferriman-Gallwey (m-FG) score up to 9–10 is considered normal (27). In our study, 59 patients in the anovulatory group fell into a diagnostic “grey zone,” with total testosterone levels between 40 and 50 ng/dl and/or an m-FG score between 8 and 16. The use of a testosterone cut-off (50 ng/dl) during patient selection likely explains why the LH/FSH ratio showed low sensitivity but good specificity in our cohort. Long-term follow-up of these patients may help determine more accurate testosterone thresholds for diagnosing PCOS in adolescents.

Obesity and insulin resistance are frequently associated with menstrual irregularities in adolescents, and PCOS is uniquely characterized by disturbances in both insulin and lipid metabolism. In a study by Green et al., non-obese adolescents with PCOS exhibited greater insulin resistance and hepatic fat accumulation compared to healthy controls, despite lower caloric intake (1,379 kcal/day vs. 1,577 kcal/day). The authors attributed these metabolic alterations to muscle mitochondrial dysfunction (28). Furthermore, PCOS is associated with an 18-fold increased risk of developing diabetes, with prevalence rates of 16% in non-obese and 50%–70% in obese patients (29). Similarly, in our study, approximately one-quarter of PCOS patients exhibited insulin resistance. A meta-analysis by Li et al., involving 13 studies and 756 patients, found that obese adolescents with PCOS had higher levels of total and free testosterone, fasting insulin, HOMA-IR, fasting glucose, leptin, and lipid profile abnormalities, alongside lower SHBG levels, compared to normal-weight PCOS patients. Interestingly, similar differences were observed between obese adolescents with and without PCOS, suggesting that obesity exacerbates the clinical severity of PCOS (29). In our cohort, adolescents with PCOS had significantly higher total testosterone and DHEA-S levels, as well as lower BMI and HOMA-IR values, compared to hyperinsulinemic/obese adolescents with oligomenorrhea. These findings indicate that ovarian dysfunction, rather than hyperinsulinemia, is the primary driver of hyperandrogenemia in PCOS. While hyperinsulinemia exacerbates androgen production via LH sensitivity in theca cells FOH is the central mechanism underlying PCOS pathogenesis (28, 30, 31).

The FAI has been proposed as a diagnostic tool for adolescent PCOS in recent studies. Sağsak et al. reported that an FAI cut-off >6.15 demonstrated 89% sensitivity and 77% specificity in adolescents (32). Similarly, Khashchenko et al. identified an FAI cut-off >2.75, with 75% sensitivity and 93% specificity, in a study of 130 adolescents with PCOS and 30 healthy controls (26). Yetim et al. also found significantly elevated FAI levels in adolescents with PCOS compared to controls (6.7 vs. 3.0) (33). However, the interpretation of FAI values remains challenging due to the complex interplay between SHBG and factors such as hyperinsulinemia, obesity, diabetes, and hypothyroidism. Obesity, which disrupts glucose-insulin metabolism, is associated with decreased SHBG levels (34). Bideci et al. previously reported significantly lower SHBG levels in obese PCOS patients compared to non-obese PCOS patients (30). In Khashchenko et al.'s study, adolescents with PCOS had a significantly higher BMI than healthy controls (*p* = 0.0002) and, despite the lack of postprandial glucose and insulin data, exhibited higher leptin levels. Similarly, Yetim et al. found that the mean BMI *z*-score of adolescents with PCOS was higher than that of healthy controls (1.4 vs. 0.8) (33). In our study, we compared PCOS patients to hyperinsulinemic/obese adolescents to assess the impact of hyperinsulinemia and obesity on FAI and SHBG. We found that hyperinsulinemic/obese adolescents had slightly higher FAI values (8.30 vs. 8.0) and significantly lower SHBG levels (12.2 vs. 29.2 nmol/L) compared to PCOS patients. These results suggest that the use of FAI as a diagnostic criterion for PCOS may be problematic, particularly in populations with obesity and insulin resistance. Therefore, the diagnostic utility of FAI in adolescents should be approached with caution. The application of the Rotterdam criteria in adolescents remains controversial, particularly regarding the assessment of ovarian cyst counts. The high prevalence of anovulatory cycles and the physiological presence of multiple ovarian follicles (up to 24) during adolescence complicate this diagnostic approach. Additionally, the challenges associated with performing transvaginal ultrasonography (USG) and pelvic MRI in adolescents further limit its practicality. In prior studies, ovarian volume has emerged as a more reliable diagnostic marker, with thresholds of 15 cc in a single ovary or 12 cc in both ovaries proposed for this population (7, 10, 35, 36). Our findings support this, as suprapubic ultrasonography revealed a mean ovarian volume of 11.8 cc in PCOS patients, reinforcing the reliability of ovarian volume as a diagnostic criterion in adolescents.

In the girls classified as having anovulatory cycles (*n*: 229), no specific underlying cause was identified. These findings raise important considerations regarding the long-term risk of developing PCOS in adolescents presenting with anovulatory cycles. These patients did not fulfill the diagnostic criteria for PCOS at the time of evaluation. However, more than 50 girls in this group fell into a diagnostic gray zone, with borderline serum testosterone levels (40–50 ng/dl) and borderline hirsutism scores (modified Ferriman-Gallwey score of 8–16). Considering these findings, and according to the latest definitions, these girls with anovulatory cycles may be considered “at risk” for PCOS and would require long-term follow-up up to 8 years post gynecological age—to establish a confirmatory diagnosis of PCOS.

This study has several limitations. First, the retrospective design limited control over confounding variables and restricted the depth of data collection, particularly regarding longitudinal follow-up. Second, being conducted at a single tertiary care center may affect the generalizability of findings to broader populations. Third, 59 patients fell into a diagnostic gray zone with borderline testosterone levels (40–50 ng/dl) and hirsutism scores (modified Ferriman-Gallwey 8–16), emphasizing the challenge of applying strict diagnostic cut-offs in adolescents where physiological variations can mimic PCOS. Despite these limitations, the study provides valuable insights into the hormonal and etiological features of PCOS in adolescents. Key strengths include the large cohort (*n* = 305), the use of standardized, adolescent-specific diagnostic criteria, and a multidisciplinary clinical evaluation involving four pediatric endocrinologists and an adolescent health specialist. To our knowledge, this is the first study to specifically differentiate between PCOS and anovulatory cycles in adolescents presenting with oligomenorrhea.

We conclude that current diagnostic tools, particularly the FAI and SHBG, demonstrate limited specificity in distinguishing PCOS from other causes of anovulation in adolescents. Although the LH/FSH ratio exhibits limited sensitivity, clinical or biochemical evidence of hyperandrogenism remains the most reliable diagnostic indicator in this age group. Additionally, increased ovarian volume is a consistent ultrasonographic marker. Our findings suggest that reliance on a single biomarker is inadequate for diagnosing PCOS in adolescents. A multiparametric approach, incorporating the LH/FSH ratio, SHBG, FAI, and ultrasound assessment of ovarian morphology, may enhance diagnostic accuracy. However, confounding factors such as obesity, hyperinsulinemia, and nutritional status must be carefully considered when interpreting endocrine markers. To address these challenges, future research should prioritize large-scale, prospective, multicenter studies with standardized diagnostic protocols to validate and refine PCOS criteria for adolescents. Clinically, developing adolescent-specific diagnostic algorithms that account for pubertal status and metabolic risk factors could improve early diagnosis and guide effective management.

## Data Availability

The raw data supporting the conclusions of this article will be made available by the authors, without undue reservation.
